# The political sociologist Seymour M. Lipset: Remembered in political science, neglected in sociology

**DOI:** 10.1080/23254823.2019.1570859

**Published:** 2019-02-26

**Authors:** Philipp Korom

**Affiliations:** Department of Sociology, University of Graz, Graz, Austria

**Keywords:** Seymour M. Lipset, reception studies, political science, sociology, citation analysis, scientometrics

## Abstract

Seymour M. Lipset was one of the leading scholars of his generation in political science and sociology. Empirically, this article highlights divergent receptions of Lipset in both disciplines: while Lipset experiences increasing visibility in political science, his visibility in sociology turns out to be rather transitory. To better understand these divergent legacies, this paper identifies major topics in Lipset’s oeuvre and reconstructs the ideas that received the most attention in both disciplines since the 1950s. It becomes apparent that the demise of Lipset in sociology can be explained through his decreasing impact on social stratification research and the fact that three major contributions, *Political Man, Party Systems and Voter Alignment,* and *Some Social Requisites of Democracy,* never entered the sociological canon. In contrast, Lipset’s work on democracy has gained a strong foothold in political science and his groundbreaking ‘cleavage theory’ is of continuing relevance to political scientists.

## Introduction

Seymour M. Lipset (1922–2006) was among the most important sociologists of his generation. His work mostly revolved around political topics. His main publications facilitated the establishment of the subfield ‘political sociology’ (Janoski, 2005),^1^ and he was the leading figure behind the foundation of the international *Research Committee on Political Sociology*.^2^ That most of his work stood at the crossroads between sociology and political science reflects the fact that he was the first person to serve as president of the *American Political Science Association* (1979–1980) and the *American Sociological Association* (1992–1993). Lipset (1986) himself argued that the reason his book, *Political Man*, is commonly ranked in rosters of the most-cited works in the social sciences rests not only on its availability in many languages but also in its interdisciplinary orientation.^3^

Lipset’s citation impact has often been the source of much commentary. It has been, among other things, posited that ‘no living political scientist or sociologist is more frequently cited’ (Diamond & Marks, 1992, p. 3), and one of his mentors, the sociologist Robert K. Merton, reported that ‘of the nearly 3 million scientific authors cited in the SCI […] only 3 in 10,000 have had their work drawn upon as often’, which would suggest Lipset to be ‘one of the truly consequential social scientists of our time’ (Merton, 1992, pp. x–xi).

**Figure 1.**
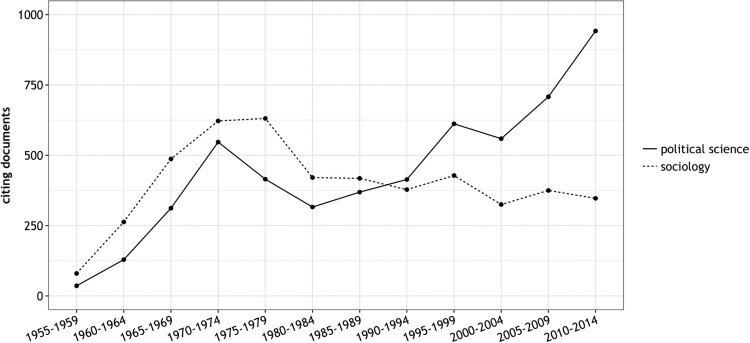
Documents citing Lipset in political science and sociology journals (SSCI, 1955–2014). Notes. The cited author search combines eleven different variants of Lipset using the Boolean OR operator (e.g. LIPSET SEYMOUR, LIPSET SM, LIPSET S).

What these commentators do not pay attention to was the discipline-specific reception of Lipset’s work. If one takes citations as an indicator for research impact, two distinct legacies become apparent (see Figure 1): in sociology, the Lipset reception reached something of a zenith in the 1970s and then declined continuously over half a century; in political science, more and more scholars started to borrow ideas from Lipset after a decline in attention between 1975 and 1985. While Lipset received nearly equal attention in both disciplines between 1970 and 1974, he is cited almost twice as often in political science than in sociology since 2000.

While a ‘late blooming’ of Lipset’s seminal article *Some Social Requisites of Democracy* is already well-documented for political science (Sigelman, 2006), the rather diametrically opposed reception trends outlined in Figure 1 pose questions that still need to be answered. How are we to explain the gradual demise of Lipset in sociology? How is it possible that the importance of a scholar rises steeply in one social science discipline while it declines in another one?

To understand the academic success of selected authors, previous research has explored commemorative practices (Gill, 2013); the places, actors and outlets through which an author spread his ideas (Lamont, 1987); the various flows of translations that helped garner a global dominance (Santoro, Gallelli, & Grüning, 2018) or the appropriation of key concepts that stay closely associated with an author’s name (Sallaz & Zavisca, 2007). This article takes yet another analytical approach by comparing receptions within two different social science communities to understand key factors that lead to enduring, rather than transitory, scholarly eminence.

The case of Lipset appears to be particularly suited for such an endeavour because of the hybridity and vastness of his work. Lipset crossed disciplinary boundaries between political science and sociology so frequently that it is unclear where he belongs in the wider pantheon of social scientists. Furthermore, his oeuvre is multi-faceted, covering such different topics as social mobility research and student politics. Given the veritable cornucopia of academic contributions, it is rather unpredictable which ideas ‘survive’ in the long run. At the risk of oversimplification, and given the insights gained in Figure 1, two major scenarios may be imagined. Either sociologists and political scientists borrow the same (scenario 1) or different ideas (scenario 2), as the attention given to Lipset increases in political science while it decreases in sociology.

To probe which of these idealised scenarios fits the real world better, this article proceeds in three steps. So that we can try to establish a fair overview of the disciplines we are discussing, our methodology seeks to be as neutral as possible among competing theoretical positions. First, we empirically establish the major topics that run like a red thread through Lipset’s work and link every publication to one or several of these topics. Second, we quantify the attention paid in each discipline to selected publications and the collective work on a given topic (for example, American exceptionalism) by counting citing articles in academic journals. By doing so, we detect the ‘multiple lives’ of Lipset in the literature (Ollion & Abbott, 2016). Third, we apply detailed citation context analysis to determine which key ideas are borrowed from Lipset by both political scientists and sociologists.

## Introducing Seymour M. Lipset

### Lipset’s career in academia

A gloss on the biography and the academic contexts in which Lipset developed his many accomplishments appears necessary to understand this versatile scholar. Lipset was born in New York City in 1922 into a working-class family of East European Jewish immigrants.^4^ At Townsend Harris High School, a preparatory school for City College, he became involved in the youth group of the Socialist Party, the Young People’s Socialist League (YPSL).^5^ At City College of New York, the student Lipset regularly engaged in lunchroom meetings dedicated to exploring Marx’s writings.

In the fall of 1943, Lipset started his studies at Columbia University. Columbia was already a major centre for graduate education in sociology when Robert K. Merton arrived in 1941, and with Paul F. Lazarsfeld’s appointment, it became the most influential base for Ph.D. training. In his own brief memoir, the article-length ‘Steady Work’, Lipset describes the sociologist Merton as the ‘most important intellectual influence’ (Lipset, 1996, p. 7). Columbia sociology combined the theorising of Merton with the methodological expertise of Lazarsfeld (Coleman, 1990). Its ‘research laboratory’ was the Bureau of Applied Social Research (BASR). The many BASR large-scale social research projects provided students with research experience and made data available to them. Further, the BASR became a fertile breeding ground for a sub-discipline that was then still in its infancy – political sociology (Glock & Sills, 1958).

Lipset received a fellowship from the Social Science Research Council (SSRC) to study the Cooperative Commonwealth Federation (CCF) of Saskatchewan for his doctoral dissertation (1945–1946). He worked as a lecturer at the University of Toronto (1946–1948), defended his dissertation in spring 1948 and accepted an associate professorship position at UC Berkeley (1948–1950), which enabled him to access – together with Reinhard Bendix – massive job history data gathered by the Institute of Industrial Relations, which was based in Oakland, California. Merton invited Lipset back to Columbia with an associate professor position (1950–1956), which resulted in a period of intense cognitive interaction between the onetime-student turned professor, Lipset, and many talented students (including Martin Trow or James S. Coleman). From the 1950s, he travelled frequently to Europe and other parts of the world, becoming an intellectual known beyond the confines of the United States (Velasco, 2004, p. 588). In 1956, Lipset spent a year together with his research assistant, Juan Linz, at the Center for Advanced Study in the Behavioral Sciences (CASBS) in Palo Alto, for a research project about the social bases of political diversity. This cooperation led to a co-authored book that was never published but furnished the ground for *Political Man* (Linz & Lipset, 1956).

He continued his career as a full professor at UC Berkeley (1956–1965), where he renewed his cooperation with Bendix. In 1965, he moved to Harvard University (1965–1975) mostly to free himself from the time-consuming involvement in Berkeley academic politics (Lipset, 1996, p. 17).^6^ At Harvard, he established an amicable relationship with Talcott Parsons (Velasco, 2004, p. 594) and mentored the student Theda Skocpol. In 1975, Lipset left Harvard and became a professor at Stanford University, where he began to work at the Hoover Institution. Having reached mandatory retirement age, Lipset continued to work as a Professor for Public Policy at George Mason University (1992–2001).

Admittedly, this is a short exposition of Lipset’s remarkable academic career.^7^ He is remembered by one of his students as ‘the most work-focused and hardest working person I encountered in a variety of elite institutions’ (Marx, 2006, p. 78). As Hoffmann-Lange (2009) has noted, he collaborated with scholars, such as James S. Coleman or David Riesman, who defy a simple disciplinary classification; he mentored younger colleagues who either became renowned sociologists (such as Ann Swidler) or political scientists (such as Juan Linz); and he served as a professor of political science *and* sociology (for example, at Stanford University). Thus, Lipset can be considered one of the true figureheads of the hybrid ‘political sociology’.

### Lipset’s career in print

**Figure 2.**
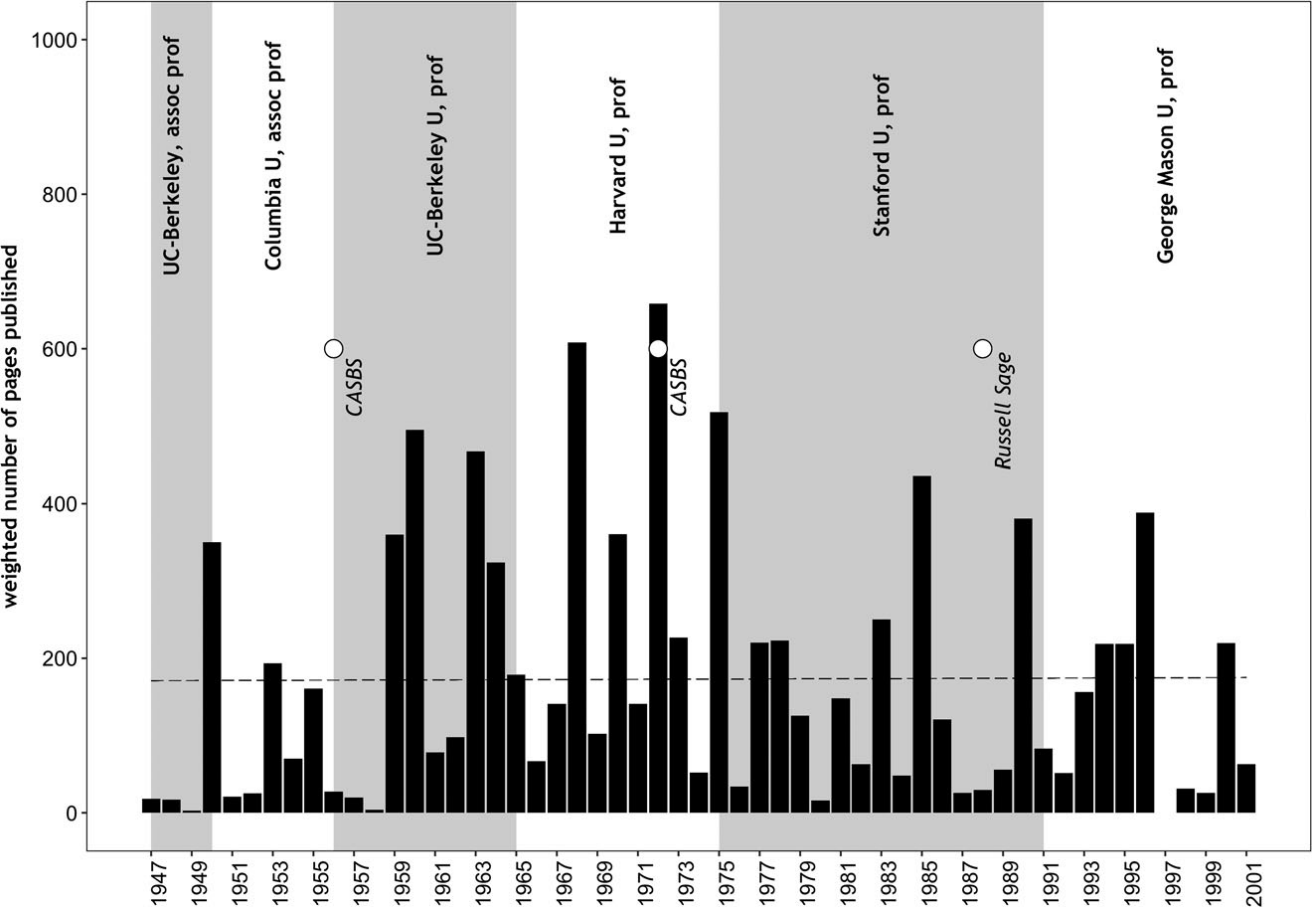
Weighted page count for published monographs, book contributions and journal articles (1947–2001). Notes. Considered are 23 books (including one research report), 139 book contributions and 103 journal articles listed either in the appendix of Marks and Diamond (1992), in Horowitz (2003) or in Lipset (1996). Neglected are edited books and pamphlets. The page count was weighted by simply dividing for each item the published pages by the number of co-authors. The average number of published pages per year is 175 (dashed line). Circles stand for research stays of at least one year; the abbreviation CASBS stands for the Center for Advanced Study in Behavioral Sciences at Stanford University.

A central characteristic of Lipset the academic is his outstanding life-long productivity. Figure 2 delineates his career in print by using the (weighted) number of published pages per year. While the trajectory is curvilinear, reaching its peak in the early 1970s, it becomes clear that Lipset published at least 1000 pages every ten years between 1947 and 2001.

His first book, *Agrarian Socialism* (Lipset, 1950), resulted from his dissertation and aimed to explain the rise to political power of the social democratic Co-operative Commonwealth Federation (CCF) in the Canadian province of Saskatchewan. Thus the young leftist from New York hoped to better understand why his own country had never produced a serious socialist movement. Six years later, Lipset published the landmark study *Union Democracy* (Lipset, Trow, & Coleman, 1968), in which the ITU was found to be a significant exception to Michel’s ‘iron law’. In his last book, *It Didn’t Happen Here* (Lipset & Marks, 2000), in which he addresses the question ‘Why is there no socialism in the United States?’ Lipset returns to the topic of ‘American exceptionalism’ to which he had previously dedicated much of his research. Between *Agrarian Socialism* and *It Didn’t Happen Here,* Lipset wrote another 20 books on different subjects, ranging from student politics or social mobility to change and continuity in the American Jewish community. Some of the books, such as *Political Man* (Lipset, 1981) or *Revolution and Counterrevolution* (Lipset, 1968), are collections of previously published journal articles or book chapters.

While monographs make up approximately 5000 pages (if divided by the number of co-authors), book contributions amount to approximately 3000 pages.^8^ With approximately 1400 pages of articles published in 53 different academic journals, this publication genre contributes the least to the overall page count.

As ‘Lipset wrote [literally] about everything’ (Lakin, 2011, p. 337), the wealth of his research contributions can hardly be overlooked. Scholars, however, have identified the following major themes in his thought. For Diamond and Marks there lies a single core theme to Lipset’s work: ‘the conditions, problems, dynamics, values and institutions of democracy, both in the United States and comparatively throughout the world’ (Diamond & Marks, 1992, p. 3). For Laslett (1983), there are two interconnected red threads: ideas related to American ‘exceptionalism’, discussing how far, and in what respects, the United States differs from other advanced industrial societies; the concern is to specify the conditions for a stable democracy. Velasco (2004) adds to this list of topics the extreme right; Marx (2006) adds Jewish identity and social mobility; Lakin (2011) adds authoritarianism and working-class politics; and Fischer and Swidler (2016) add Canada, the labour movement and social class. Lipset (1996) himself felt that the politics of academics and intellectuals informed a major part of his research agenda through much of the 1970s.

Thus, when asked to find the essentials in Lipset’s work, different scholars offer different classification grids. To clarify more precisely which topics dominate Lipset’s writings, we apply automatic text analysis to his oeuvre to empirically establish on which of these topics Lipset worked most and how and when his research interests changed over time. The simple assumption underlying this quantitative methodology for identifying distinct research domains is that an author uses different vocabulary when writing on only loosely related topics.

## Identifying central research topics

### Exploring Lipset’s research agenda: A simple words-as-classifiers approach

The key advantage of computer-aided rather than hand-coded content analysis is that the rules are made explicit (Weber, 1984). In our approach, words (indeed, strings of characters) are the basic unit of analysis. All we want to know is what words appear in the text and with what frequency. Texts are thus treated as unstructured ‘bags of words’, and word order and relations are ignored. The overall topics (*N* = 15) to be searched in texts are determined beforehand. Each topic is represented by a ‘dictionary’, that is a list of key words that were found to stand for certain topics. Dictionaries were ‘calibrated’ by extracting the most frequent ‘meaningful’ words from the most representative texts.^9^ The main drawback of the chosen ‘dictionary method’ (Grimmer & Brandon, 2013) is that it relies to a non-trivial degree on the judgements of the analyst who decides, among other things, how many and which ‘dictionaries’ should be included. However, the selection of topics in the current study was not arbitrary but based on secondary literature that exists on Lipset’s work. Moreover, all dictionaries were developed through a close reading of Lipset’s complete work. Both factors should have reduced the subjectivity of the researcher in the application of this methodology.

Dictionaries contain, with one exception, between 20 and 30 words (see Online Appendix). The computer programme used, *MAXQDA,* ignores different word endings and capitalisation. For example, the dictionary entry ‘jew’ suffices to count the frequencies of such different words as ‘Jew’, ‘Jewish’, ‘Jewry’ or ‘Jewishness’.

To assign contributions to topics, we first delete 310 extremely common stop-words – that are of little value for our analysis – from all texts and then calculate the percentage ratio of dictionary-based keywords to total number of *n*-grams (*n* > 2) contained in each publication.^10^ If the ratio is above 10 percent, we accept the ‘match’.

**Figure 3.**
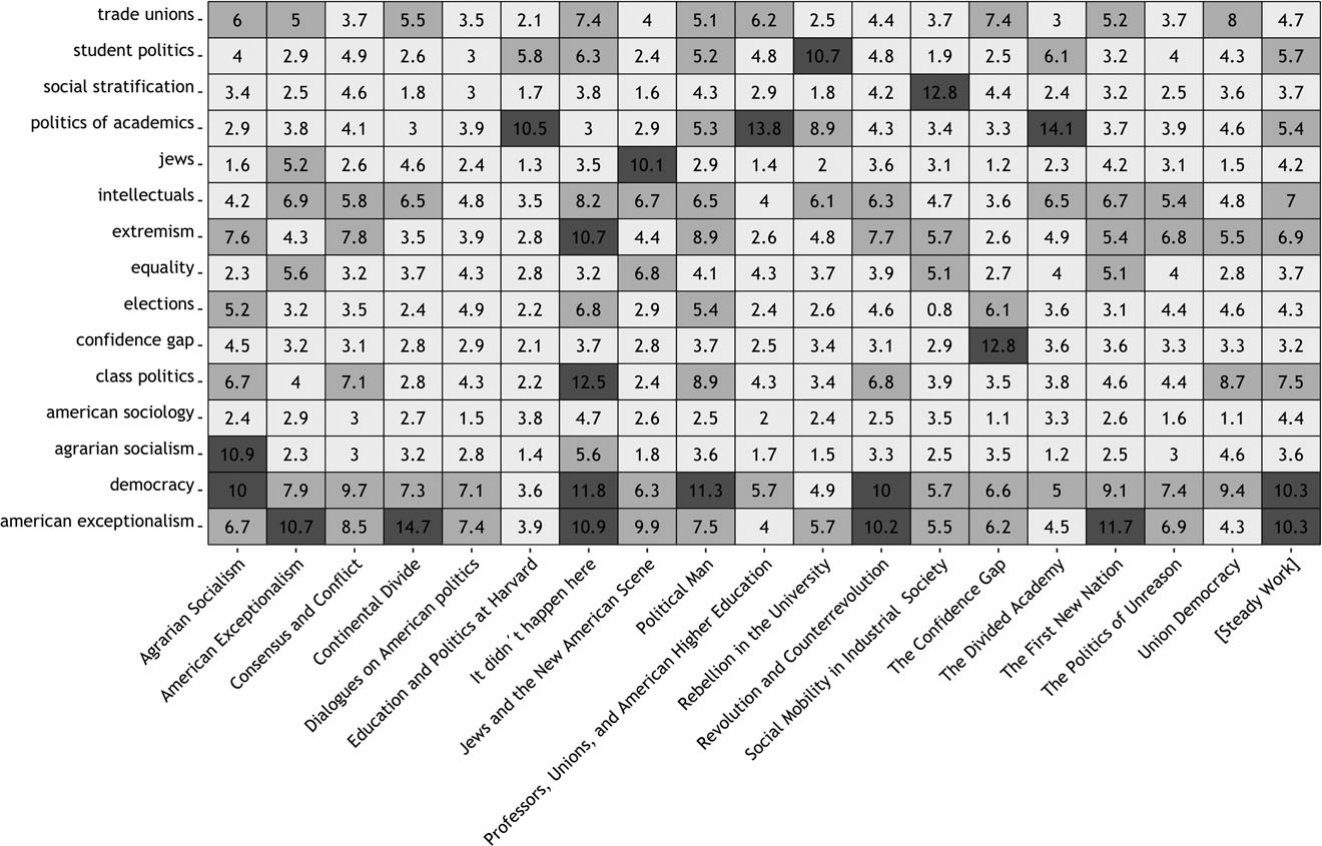
Topics scores of 18 books (in %). Notes. Considered are all books listed in the Online Appendix A1. Percentage numbers indicate the proportion of text in each book that is covered by dictionary-specific key terms. The title ‘[Steady Work]’ refers to Lipset’s academic memoir published in the Annual Review of Sociology.

While we calculated the ‘fit’ between each publication and each ‘dictionary’, for the purposes of demonstration, we present here only the results for all books and Lipset’s academic memoir. To construct Figure 3, occurrences for each dictionary entry within all considered books and the memoir were counted. The output of the analysis consists of values for each ‘dictionary’/topic, represented as a percentage of the total words contained in one of Lipset’s 19 selected contributions. To give an example: all words of the dictionary ‘student politics’ cover 10.7 percent of all words in the book ‘Rebellion in the University’ after the deletion of 310 stop-words. Dark fields in Figure 3 indicate all ‘matches’ between books and topics.

As Figure 3 shows, most books load on a single topic dictionary with two notable exceptions. Three publications are assigned to ‘American exceptionalism’ as well as to ‘democracy’, suggesting that these topics are intertwined. This is confirmed by the finding that Lipset’s (1996) academic memoir ‘Steady Work’ loads on both dimensions.

Interestingly, the content of *It Didn’t Happen Here* is even covered by four different dictionaries: class politics (that is, class differences in politics; for a definition see Weakliem and Adams (2011)), democracy, American exceptionalism, and extremism. This pattern should not surprise, as in this last book, Lipset discussed and synthesised all major explanations for American exceptionalism that have been proposed over the past century – namely, the distinctive character of U.S. political institutions, the ethnic heterogeneity of the nation’s population, the divisions between labour unions and the socialist party or the sectarianism of American socialists.

Overall, Figure 3 confirms the validity of the chosen method. There is no apparent mismatch, and the threshold of 10 percent appears to be well chosen. The main insight gained from the quantification approach is that despite a broad scope of research interests, two research topics prevail: democracy and American exceptionalism.

### Lipset’s shifting research interests over time

**Figure 4.**
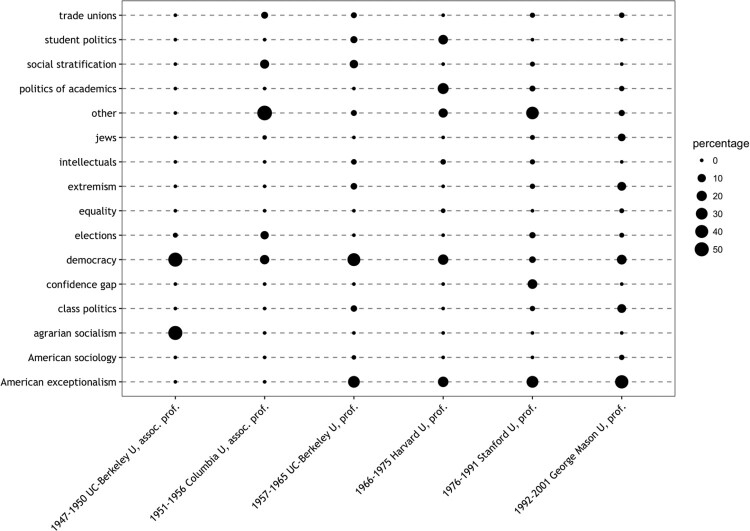
Lipset’s changing research agenda measured in number of weighted pages. Notes. Considered are all books and journal articles listed in the Online Appendix A1 that are assigned to one or more topics. The weighted pages of these publications are summed up per topic. The percentage numbers indicate the proportion of text (as measured by weighted pages) assigned to a topic for a given time episode (e.g. 1966–1975 Harvard U., prof.); percentages in each ‘column’ thus come up to 100%.

To further explore Lipset’s research, we apply the same methodology to another 92 journal articles published by Lipset (see Online Appendix), which enables us to comprehensively capture the sheer quantity of research published in different research domains.^11^ Figure 4 displays statistics for six different employment episodes between 1947 and 2001 separately, to probe to what extent Lipset’s research agenda changed over time.

The key point to take from Figure 4 is that democracy was the only research topic that Lipset continued to pursue throughout his professional life. It was also his primary research interest until moving to Harvard, when it became increasingly replaced by growing research on American exceptionalism. Social-stratification research was only on Lipset’s research agenda during the 1950s and 1960s. During the Harvard period (1966–1975), his research revolved around the issues of student politics and the politics of academics. These interests, however, vanished after a decade of intense research. While Lipset worked on the topic of agrarian socialism at the very beginning, he turned to class politics at the end of his academic career.

The fact that Lipset collaborated, closely with different co-authors (including Reinhard Bendix or Everett C. Ladd), especially in the fields of social stratification and politics of academics, results in smaller numbers in Figure 4 that depicts the *weighted* paged count. An alternative depiction based on a simple page count yields greater values for both research strands. In both approaches, however, it transpires equally that both research activities were, more or less, confined to certain career stages only.

Taking a more dynamic view on the research agenda thus reveals that Lipset’s research focus was alternating between democracy research (1947–1965) and research on American exceptionalism (1966–2001). Overall, it can be concluded that apart from these two lifelong research themes, Lipset dedicated his time and energy to various research areas (such as social stratification or student politics) but always for a limited time.

## Reconstructing the reception of Lipset in two disciplines

### The research tools: Web of Science (WoS) and JSTOR Data for Research (DfR)

In bibliometric research, the former *ISI Web of Science* is the standard and most widely used tool for generating citation data as indicators for the reception of a scholar’s work. ISI, the Institute for Scientific Information, was founded by the bibliometrician Eugene Garfield in 1960. In 1992, ISI was sold to the *Thomson Reuters* corporation (‘Thomson IS’). Today, the citation indexing service is maintained by *Clarivate Analytics*. *Thomson Reuters* had already made its citation databases accessible using the online platform called ‘Web of Science’ (previously known as ISI Web of Knowledge).^12^

We base our citations analysis on the Social Science Citation Index (SSCI-present) that is included in the WoS.^13^ The SSCI citation count used here is the *number of articles published in SSCI journals citing Lipset at least once*.^14^ Every search was not only limited to the timespan ‘1956-present’ but also to one of the two WoS categories: ‘political science’ and ‘sociology’. WoS categories are purely journal-based and non-hierarchical and were developed already by ISI staff, who started to assign journals to subject categories based on journal titles and citation characteristics and later developed sophisticated strategies to cluster semantically similar journals (Pudovkin & Garfield, 2002).

The other research tool we use to study the reception of Lipset’s work is JSTOR data for research (DfR).^15^ JSTOR, an independent not-for-profit organisation that was conceived in 1994 by William G. Bowen, then president of the Andrew W. Mellon Foundation (Schonfeld, 2003), works with diverse groups of approximately 1200 publishers to preserve and make their content digitally available. DfR provides datasets containing meta-data as well as the full contents of JSTOR items for use in research. Using DfR, we could identify 6726 journal articles in political science journals and 8315 journals articles in sociology journals mentioning the word ‘Lipset’ at least once in the title, abstract, main text or in the reference section. We used the content of these 15,041 journal articles – that do not fully overlap with the WoS text corpus analysed – to identify changing topics in the reception of Lipset between 1947 and 2017.

### Lipset in two neighbouring disciplines: Evidence from the SSCI

**Figure 5.**
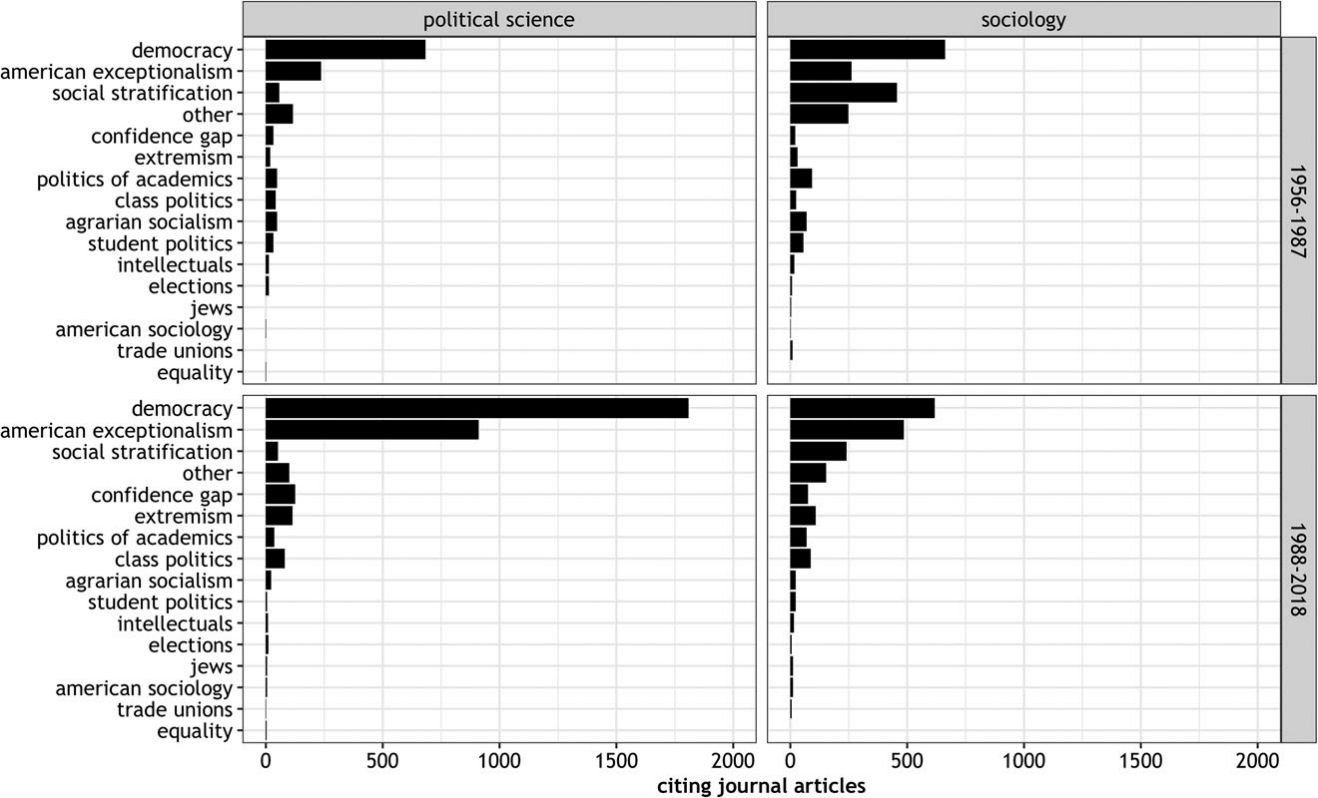
Discipline-specific reception of Lipset measured by SSCI citations. Notes. Considered is the number of journal articles citing one of Lipset’s publications listed in the Online Appendix A1. Citing articles are assigned to topic categories. To give an example: As the book ‘Political Man’ was assigned to the category ‘democracy’ all the 1501 articles citing ‘Political Man’ in political science journals are also attributed to the topic ‘democracy’.

To reconstruct the reception of Lipset in political science and sociology, we build on the previously conducted content analysis and take cues from scientometrics. Having assigned each book and journal article listed in the Online Appendix A1 to one or more topics, we can tell which research – and not only which specific publication – attracted the most attention by peers in two social sciences disciplines. Figure 5 displays the proportion of citing articles for each topic during two time episodes (1956–1987; 1988–2018).

The overall picture that emerges for each discipline overlaps to some degree. Even if the versatile scholar Lipset published in many different fields (see Figure 4), the bulk of citations in both disciplines refer to his contributions on democracy and American exceptionalism.

Over time, the reception patterns diverge strikingly. In political science, Lipset garners a substantially growing number of citations for his work related to democracy and American exceptionalism. Looking at the results for sociology, we see little change, with one notable exception. Publications on social stratification started to garner a rather average number of citations.

**Figure 6.**
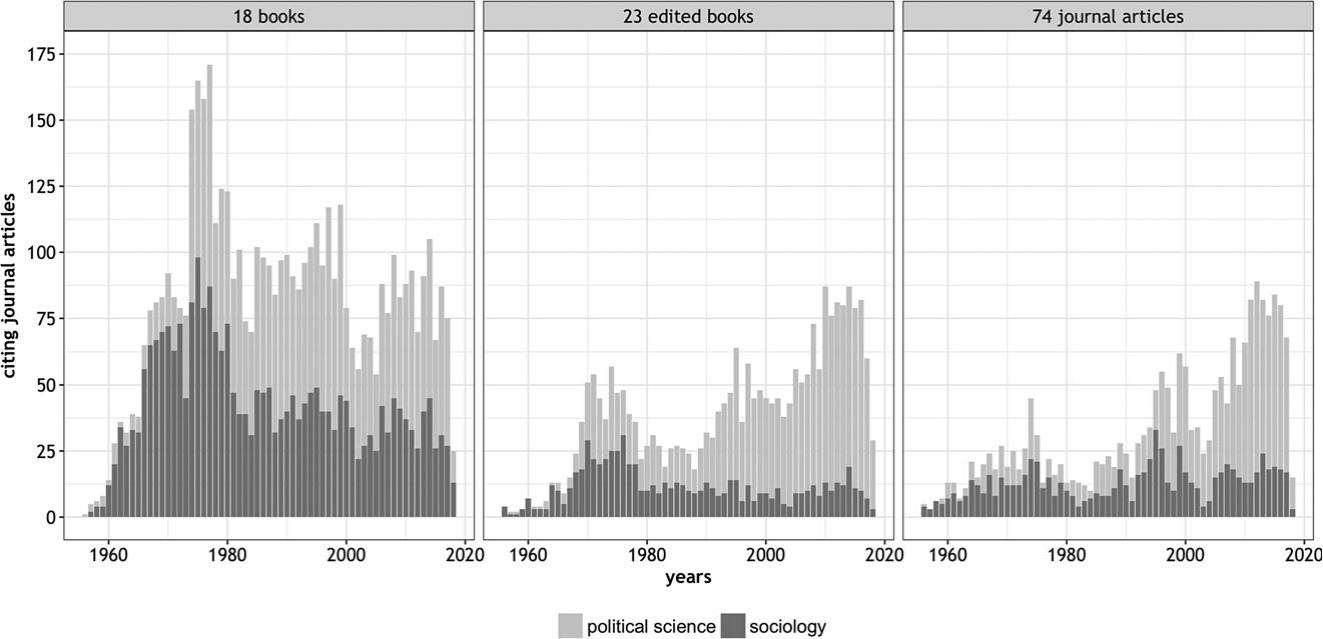
Journal articles citing Lipset by publication type and discipline (SSCI, 1956-present).

To bring more light to the divergent reception trends we disaggregate SSCI citation on the level of publication type and choose a more fine-grained temporal scale (see Figure 6). Regarding all books authored by Lipset, one can see that the number of citing articles peaks in both disciplines between 1970 and 1980 and then stabilises on a far lower level. In absolute terms, however, Lipset’s books are cited twice as much today in political science compared to sociology. With respect to edited books and journal articles, we even see clearly divergent trends. While the number of citing articles grows in political science over time, it stagnates in sociology.

If one disaggregates these citation counts (see Online Appendix) and looks at the citation trajectories of publications that contribute the most to the overall citation volume, one can easily identify the underlying reasons for the different reception histories identified.^16^

The book *Political Man* remains often-cited in political science but lost traction in sociology. Citations of the sociological classic *Social Mobility in Industrial Society* decreased. The book *Party Systems and Voter Alignment,* in which the editors, Lipset and Rokkan, outline the importance of social cleavages for the ‘freezing’ of party systems more than forty years ago, appears more relevant today to political scientists than ever. Finally, the 1959 journal article *Some Social Requisites of Democracy,* in which Lipset identified a nexus of causal factors leading from economic development to prospects for stable democracies, garners more citations every ten years in political science, while it remains rarely cited in sociology journals.

### Lipset in two neighbouring disciplines: Evidence from JSTOR

To validate the ‘citation approach’, we use a second method that aims at identifying dominant topics covered in the reception of Lipset by analysing the full content of JSTOR-archived journal articles mentioning the word ‘Lipset’ at least once. JSTOR provides up to ten ‘Topic Cards’ derived for each article from a thesaurus, which are intended to provide background and content for the many subjects covered on JSTOR. To give an example, the article ‘Some Social Requisites of Democracy’ is assigned the following topics: democracy, political parties, dictatorship, economic development, countries, social democracy, urbanisation, communism, and Catholicism.^17^

Table 1 displays only the most frequent topics for two disciplines and different time-spans. It becomes apparent that the bulk of all articles contain the following topics: democracy, political parties, liberalism, conservatism, and political attitudes. There are also discipline-specific topics. During the period 1951–1987, most articles in sociology mentioning Lipset discuss issues that either relate to social mobility or to social classes. After 1987, such topics as ‘social mobility’ or ‘working class’ cease to be ranked among the top 0.5 percent top keywords. In political science, voting behaviour, political campaigns and changing political partisanship appear to play a much greater role in the Lipset reception.

**Table 1.** Top JSTOR topics by discipline.

	Political science			Sociology		
	1947–1987	1988–2017	All years	1951–1987	1988–2017	All years
**Top 0.1%**	Political parties	*Democracy*	*Democracy*	Political parties	*Democracy*	Political Parties
	Voting	Political parties	Political parties	Prestige	Political parties	*Democracy*
	*Democracy*	Liberalism	Voting	Communities	Liberalism	Conservatism
			Liberalism	Social theories	Capitalism	Liberalism
						Political attitudes
**Top 0.5%**	Political attitudes	Voting	Political attitudes	Conservatism	Conservatism	Social theories
	Liberalism	Authoritarianism	Conservatism	Political attitudes	Political attitudes	Capitalism
	Conservatism	Conservatism	Economic development	Social structures	Voting	Communities
	Political Systems	Political attitudes	Political partisanship	*Social mobility*	Social theories	Voting
	Communism	Economic development	Authoritarianism	Liberalism	Communities	Prestige
	Political partisanship	Political partisanship	Political candidates	Universities	Men	Social structures
	Political candidates	Political candidates	Elites	Capitalism	Social movements	Universities
	Economic development	Countries	Political systems	Voting	Modeling	Working class
	Public opinion	Elites	Public opinion	Working class	Social research	Social mobility
	Socialism	Capitalism	Communism	Social interaction	Protestantism	Social interaction
		Modeling	Capitalism	Parents	Universities	Social research
			Political campaigns	Social classes	Working class	Modeling
			Presidential elections	Middle class		Parents
			Socialism	Social research		Protestantism
			Communities	Modeling		Social classes
						Educational Attainment
						Middle class
						Men
						Political Protest
						Social movements

Note: The topic terms are ordered in descending order.

## The afterlife of the most cited publications in two disciplines

It has become apparent that the case of Lipset confirms the more general observation that if an author has an afterlife in the literature, the majority of citations will typically refer to very few major works (Ollion & Abbott, 2016). While we could reconstruct citation trajectories that move either towards oblivion or canonisation and identify the few key writings that remain relevant to political scientists and sociologists, we still need to explore exactly why a few contributions receive disproportionately large attention from political scientists or sociologists.

As we can see, the book *Social Mobility in Industrial Society* stands out in sociology through its outstanding citation impact. To better assess why, even half a century after publication, this landmark work of social stratification research is, to some extent, still relevant to sociologists, we hand-coded a random sample of 100 citations between 1980–1990 and 2000–2010. As Table 2 shows, about one fifth of all considered citations are perfunctory, that is, the citation is mainly an acknowledgment that serves no explicit role in the analysis (Bornmann & Daniel, 2008). Between twenty and thirty-four percent of all other citations refer to what became to be called in the literature the ‘Lipset and Zetterberg hypothesis’ or the ‘LZ-hypothesis’.

**Table 2.** Content of *Social Mobility in Industrial Society* referred to in citing articles that are classified as belonging to sociology (in %).

	1980–1990	2000–2010
Classification scheme, definition, methodology	6	10
Entrepreneurship/self-employment	6	0
Homogamy	0	10
Industrialization and mobility	4	14
Lipset and Zetterberg hypothesis	34	20
*Perfunctory citation*	*16*	*20*
Religious affiliation and achievement	6	0
Social psychology of inequality	6	6
Other topics	22	20
	100	100

Note: Considered are fifty randomly selected citing articles for the timespan 1980–1990 and 2000–2010, thus a total of 100 journal articles.

Most authors refer to chapter two of the book, which was jointly written by Lipset and Zetterberg,^18^ in which results from national mobility surveys from the 1940s and 1950 are interpreted as evidence for comparable degrees of intergenerational mobility across industrialised societies. The following arguments are also commonly seen as part of the ‘LZ-hypothesis’: high rates of social mobility are not a precondition but a consequence of industrialisation; there is a one-time increase in observed mobility during the early take-off period of economic development (mobility threshold); once a certain stage of industrialisation has been attained, the overall pattern of social mobility is much the same in all Western European countries (invariance in mobility chances). While these arguments were mostly critically viewed, they appear, nevertheless, to have inspired subsequent research much more than any other topic discussed in the book (see Table 2).

*Political Man: The Social Bases of Politics* is an expansion of articles previously published in professional journals. It can be seen as a survey of political sociology and its major contributions to political science. In *Political Man*, Lipset posits that in every democracy, conflicts between social groups are expressed through the party system, which at the core represents a transformation of the class struggle (‘democratic class struggle’ in Table 3). He points out that the gulf between social classes is not the only cleavage that is expressed through political parties (‘social cleavages’). It is, nevertheless, the book’s main thread that socioeconomic differences mostly determine voting behaviour (‘class voting’). He theorises the attraction of working class voters to authoritarian views (‘working-class authoritarianism’), introduces different types of political values such as (economic) liberalism/conservatism (‘conservative and liberal attitudes’)^19^ and explores the importance of education for such attitudes (‘education’). Most importantly, Lipset analsyes the social conditions of democracies (‘social requisites of democracy’), paying special attention to economic growth (‘economic development and democracy’). Finally, *Political Man* is also cited for mostly theoretical arguments related to what became known as ‘modernization theory’ (Volpe, 2010).

**Table 3.** Content of *Political Man* referred to in citing articles that are classified as belonging to political science or sociology (in %).

	Political science		Sociology	
	1980–1990	2000–2010	1980–1990	2000–2010
Class politics/voting	2	12	0	10
Conservative/liberal attitudes	4	0	12	8
Democratic class struggle	4	0	6	4
Economic development and democracy	0	10	2	2
Education	0	2	2	10
Modernisation theory	4	8	6	10
*Perfunctory*	*10*	*6*	*18*	*22*
Political participation	14	4	2	0
(Cross-cutting) social cleavages	10	12	0	4
Social requisites of democracy	2	6	0	2
Working-class authoritarianism	2	4	10	0
Other topics	48	36	42	28
	100	100	100	100

Note: Considered are fifty randomly selected citing articles for the timespan 1980–1990 and 2000–2010 for each discipline, thus a total of 200 journal articles.

Table 3 suggests, first and foremost, that *Political Man* is a cornucopia of ideas, which is evident by the great variety of different references to the work. Nevertheless, one can draw two general conclusions with all cautiousness. First, Lipset’s core arguments are more often used in political science than sociology, where we find a higher percentage of perfunctory citations. Second, the book serves mostly as a source of reference to political scientists when considering questions related to voting behaviour, political participation, or social cleavages that affect electoral behaviour, whereas sociologists tend to cite Lipset if they are writing on values (for instance, on economic liberalism or authoritarianism).

It has been claimed that *Some Social Requisites of Democracy* (1959) is to be regarded as one of the most influential political science essays of the past half-century (Diamond, 2006). In this journal article, reproduced in *Political Man* (published in the following year), Lipset posits a list of factors that constitute the conditions, though not necessarily the causes, for democracy (‘requisites of democracy’). There exist basically two interpretations of Lipset’s theory. The wide interpretation is that the interplay of many changing social conditions (such as rising educational levels, urbanisation, industrialisation) foster a democratic culture (‘socioeconomic development’) that needs to be perceived as legitimate in order to stay firmly established (‘legitimacy’). The boiled-down version of Lipset’s argument is that there is a simple correlation between per capita income and democracy, and, indeed, Lipset argues in a central passage that ‘the more well-to-do a nation, the greater the chances that it will sustain democracy’ (Lipset, 1959, p. 75).

Table 4 clearly shows that the ‘Lipset thesis’ as discussed in the literature of both disciplines tends to take the form of a rather simple formula according to which democracy is related to the state of economic development.

**Table 4.** Content of *Some Social Requisites of Democracy* referred to in citing articles that are classified as belonging to political science or sociology (in %).

	Political science		Sociology	
	1980–1990	2000–2010	1980–1990	2000–2010
Economic development and democracy	32	44	36	52
Education-democracy hypothesis	0	6	4	4
Legitimacy	8	2	0	4
*Perfunctory*	*20*	*10*	*12*	*8*
Political culture/values and democracy	0	2	0	12
Requisites of democracy	4	12	0	0
Socioeconomic development and democracy	14	6	24	12
Other topics	22	18	24	8
	100	100	100	100

Notes: For sociology, considered are twenty-five randomly selected citing articles for the timespan 1980–1990 and 2000–2010, thus a total of 50 journal articles. The numbers for political science are based on fifty randomly selected citing articles for the timespan 1980–1990 and 2000–2010, thus a total of 100 journal articles.

Overall, the citation context analysis reveals a mixed picture. In some cases, the reception of Lipset’s work is similar in both disciplines while in other cases the afterlife of a publication differs hugely. The best example of the later reception pattern is *Political Man*. As Lipset used statistical and historical data to demonstrate that social class is one of the chief determinants of political behaviour, the book became easily canonised in political science as a key contribution to the behavioural analysis of political systems. *Political Man* was also – at least initially – well received in sociology, which is best illustrated by the book winning the prestigious MacIver Award of the American Sociological Association in 1962. However, even if highly regarded by (political) sociologists, the book never influenced the collective understanding of sociology as a discipline to the same extent. As Da Silva and Vieira (2011) argue, the canonisation of texts hinges on three capacities of a text: to provide an exemplar to subsequent generations on how to conduct research, to serve disciplinary self-legitimisation, and to integrate the discipline. Regarding all three dimensions, *Political Man* was, much like Tocqueville’s *Democracy in America,* ill-suited to become a sociological classic.

## Conclusions

This article started out by establishing what can be interpreted as a paradox. Measured in citations, the eminence of Seymour M. Lipset declined in sociology from the 1980s onwards but grew significantly in political science. To better understand this paradox, this study adopted a new research design that could be of value to reception studies in general. The analysis started out by distilling major research topics from Lipset’s oeuvre using computer-assisted content analysis. It was shown that despite Lipset’s many contributions to very different fields, the largest number of his books and journal articles were dedicated to the topic of democracy and American exceptionalism. Interestingly, Lipset wrote substantially less on topics dealing with social stratification.

It was further shown that gradients of productivity translate in Lipset’s case into similar gradients of reception in the literature. The most cited works in political science and sociology touch the two major topics identified (American exceptionalism, democracy). There is, however, one notable difference in reception between the two disciplines. Sociologists have paid significantly more attention to Lipset’s work on social stratification. Thus, the reality is more ‘messy’ than stylised models of reception would suggest, and neither scenario 1, political scientists and sociologists borrow the same ideas, nor scenario 2, political scientists and sociologists borrow different ideas, fully apply.

The central finding is that there are different citation trajectories in Lipset’s work. Key publications, such as *Political Man*, *Some Social Requisites of Democracy* or *Party System and Voter Alignment*, received constant or even increasing attention over the years in political science, while garnering an ever-decreasing number of citations in sociology. A major explanation of the puzzle identified is thus that *a small number of Lipset’s major contributions* experience an ‘episodic rebirth’ (Ollion & Abbott, 2016) in political science only. The classic *Political Man* remains a source of inspiration for political scientists. In the same vain, social-cleavage theories do not only remain associated with Lipset but experience various reformulations in political science, even more so after the publication of *Party Systems and Voter Alignment*.^20^

In contrast, a key contribution to Lipset’s reputation in sociology, namely *Social Mobility in Industrial Society*, is cited because of the so-called ‘Lipset and Zetterberg hypothesis’ only. The LZ-hypothesis might today still inspire some sociological research, but scholars do not try to resuscitate a ‘Lipsian perspective’ in analyzing the social world when citing this work. Additionally, it is evident that Lipset never gained a lasting canonical status in a key knowledge domain of sociology, namely, social stratification research – a claim that we confirmed by studying changing topics in a huge JSTOR text corpus.

One must additionally consider that Lipset’s best contributions, such as *Political Man,* have not been included in the sociological canon to the same extent as, for example, Daniel Bell’s *The Coming Crisis of Post-Industrial Society* or Peter Blau’s and Otis D. Duncan’s *The American Occupational Structure*.^21^ While there may be various factors at play as to why *Political Man* never received much sociological commemoration, much suggests that – all protestations about the necessity for interdisciplinary understanding to the contrary – sociologists found the book too focused on political issues to become a sociological classic. This is not without a certain irony, given that Lipset wrote in the foreword to *Political Man* that ‘the study of man in society cannot fruitfully be compartmentalized according to substantive concerns’ (Lipset, 1981, p. IX).

More generally, this case study highlights the interplay of two central factors that determine the reception of an author. The first factor is the *originality and timelessness of ideas*. While Lipset’s early work on social mobility has proved to be outdated, his ideas on the social bases of political cleavages, for example, remain relevant. The second factor is the *ever-changing trajectories of a discipline*. Many observers of sociology, including Lipset (1994) , consider that sociology has become one of the most fragmented social-science disciplines. Research production has become predominantly organised at the levels of specialty and research area without direct reference to, or influence from, the discipline as a whole (Daipha, 2001). Furthermore, it is commonly argued that the boundaries of sociology ‘have become […] too narrow and too rigid while the bridges that link it […] to other disciplines and areas of concern may have become […] too few and insufficient’ (Aiken, 1981, p. 447). Much suggests that such an ever-increasing fragmentation and specialisation inhibit a broad reception of Lipset’s interdisciplinary and multi-faceted work within sociology. Consequently, Lipset has mutated from a central figure of sociology into a figurehead of one of the many special sociologies, namely ‘political sociology’.

Moreover, democracy research has not been able to establish itself within sociology, while it has become a major research area within political science. Especially with the ‘third wave of democratisation’ and the rise of populist parties in many established democracies, Lipset’s groundbreaking ideas on democratic legitimacy or on the relevance of socio-economic cleavages for structuring party systems have experienced a revival within political science. Lipset, the democracy theorist, thus continues to inform the social sciences, albeit mostly within the confines of political science.

## Supplementary Material

Supplemental Material

## Notes

1 In an early publication, Bendix and Lipset draw demarcation lines between political science and political sociology in the following way: ‘Political science starts with the state and examines how it affects society, while political sociology starts with society and examines how it affects the state’ (Bendix & Lipset, 1957, p. 87). Crucial differences between political sociology and political science are elaborated in detail by Sartori (1969).

2 Lipset co-founded and chaired the joint *Research Committee on Political Sociology* of the *International Political Science Association* (IPSA) and ISA (*International Sociological Association*) between 1960 and 1970.

3 *Political Man* is reported to have been sold more than 400,000 copies in 20 different languages (Marks, 2007). After having garnered more than 1400 citations it was included into the list of ‘citation classics’ by the Institute of Scientific Information (ISI).

4 His father was a Jewish Russian immigrant who worked as a printer and was a member of the International Typographical Union (ITU). Lipset grew up surrounded by talk of trade union issues and accompanied his father to union meetings at a local high school (Coker, 2002, p. 28).

5 Lipset reports that he dropped out of YPSL before leaving high school ‘as a result of disillusionment with the several factional fights’ between ‘Trotskyists and near-Trotskyists’ (Lipset, 1970, p. 144).

6 Lipset first came to Harvard in 1965 as a visiting professor. Some assume that he stayed in Harvard because of a distaste for the student revolt at Berkeley on which he conducted research (Lipset & Wolin, 1965). However, the fact that he was already negotiating with Harvard prior to the revolt speaks for a broader motivation for his decision to leave Berkeley (Schwartz, 1992).

7 For Lipset as a public intellectual, his leadership roles in Jewish organisations as well as for his partly changing political inclinations, see: Diamond (2007), Romalis (1972), Schwartz (1992), Schwartz (2017), Velasco (2004). It should also be mentioned that Lipset was also a public figure who presented his research to heterogeneous audiences through television appearances, public lectures and articles in popular journals.

8 Editorship of such monographs as *Class, Status, Power* (Bendix & Lipset, 1966) or *Party Systems and Voter Alignments* (Lipset & Rokkan, 1967) clearly also contributed to his rising academic eminence.

9 Non-keyword terms that are substantively uninteresting such as, ‘and’, ‘to’, or ‘is’ were ignored.

10 In linguistics, a n-gram is a contiguous sequence of n items. Examples for 2-grams are ‘13’, ‘to’, or ‘as’.

11 The assignment of topics to publications is made transparent in Online Appendix A1.

12 https://webofknowledge.com/

13 WoS suffers from some data quality problems that require serious attention. While every entry in the Cited Reference Index contains enough information to be uniquely identified, the cited references are not always unified; in other words, the same reference in two different records can appear differently in the database. The issue of cited reference variants can, however, be solved for authored or edited books and journal articles, but not for book contributions, which must therefore be left out.

14 It is important to note that such a measure differs from the one applied in Figure 1 that considers, as well, citations in book reviews, conference proceedings, and so on. Furthermore, one must bear in mind that WoS ignores multiple citations within texts. Both factors reduce the total number of citations considerably.

15 https://www.jstor.org/dfr/results

16 Detailed analyses on citation trajectories for the most cited publications are available from the author.

17 While information on the JSTOR thesaurus is not available, the procedure behind the assignment of topics is well documented. The application of a topic is triggered if a term contained in the JSTOR thesaurus is present at least three times in an article; the relevance is determined by how frequently the term appears.

18 Zetterberg is only mentioned in the footnote.

19 According to Lipset, if liberalism is conceived as a preference for economic redistribution, the working class is more liberal than the middle class. If liberalism is viewed as tolerance and respect for individual liberty, however, the working class is less liberal than the middle class (hence his thesis of working-class authoritarianism).

20 For the most recent reformulation of Lipset’s (and Rokkan’s) cleavage theory see Piketty (2018).

21 See, for example, the list of key works in sociology in one of the leading textbooks (Giddens, Duneier, Appelbaum, & Carr, 2009).
