# Supplementary material for: The political sociologist Seymour M. Lipset: Remembered in political science, neglected in sociology
Source: Eur J Cult Polit Sociol. 2019 Feb 26;6(4):448–73. doi: 10.1080/23254823.2019.1570859 (PMC7099882; doi:10.1080/23254823.2019.1570859)
Supplement: Supplemental Material [file RECP_A_1570859_SM8901.docx]

**ONLINE APPENDIX**

**A1. Text corpus used for content analysis (5884,7 weighted pages)**^[[1]](#footnote-1)^

*books (in chronological order); 4630,2 weighted pages*

1. Lipset, Seymour Martin. *Agrarian Socialism*. Berkeley and Los Angeles: University of California Press, 1950 [*agrarian socialism/democracy*]
2. Lipset, Seymour Martin, Martin Trow, and James C. Coleman. *Union Democracy: The Internal Politics of the International Typographical Union*. New York: The Free Press, 1968 (originally published in 1953) [*other*].
3. Lipset, Seymour Martin, and Reinhard Bendix. *Social Mobility in Industrial Society*. New Brunswick: Transaction Publishers, 1992 (originally published in 1959) [*social stratification*].
4. Lipset, Seymour Martin. *Political Man: The Social Bases of Politics*. Baltimore: The Johns Hopkins University Press, 1981 (originally published in 1960) [*democracy*].
5. Lipset, Seymour Martin. *The First New Nation: The United States in Historical and Comparative Perspective*. New York: Norton, 1979 (originally published in 1963) [*American exceptionalism*].
6. Lipset, Seymour Martin. *Revolution and Counterrevolution: Change and Persistence in Social Structures*. New York: Basic Books, Inc., 1968 [*American exceptionalism/democracy*].
7. Lipset, Seymour Martin, and Earl Raab. *The Politics of Unreason: Right-Wing Extremism in America, 1790-1970*. Chicago: University of Chicago Press, 1978 (originally published in 1970) [*other*].
8. Lipset, Seymour Martin. *Rebellion in the University*. Boston, Mass.: Brown and Co., 1972 [*student politics*].
9. Ladd, Everett K., and Seymour Martin Lipset. *Professors, Unions, and American Higher Education*. Washington, D.C.: American Enterprise Institute for Public Policy Research, 1973 [*politics of academics*].
10. Ladd, Everett K., and Seymour Martin Lipset. *The Divided Academy: Professors and Politics*. New York: McGraw-Hill, 1975 [*politics of academics*].
11. Lipset, Seymour Martin, and David Riesman. *Education and Politics at Harvard: Two Essays Prepared for the Carnegie Commission on Higher Education*. New York: McGraw-Hill, 1975 [*politics of academics*].
12. Horowitz, Irving Louis, and Seymour Martin Lipset. *Dialogues on American Politics*. New York: Oxford University Press, 1978 [*other*].
13. Lipset, Seymour Martin, and William Schneider. *The Confidence Gap: Business, Labor and Government in the Public Mind*. New York: The Free Press, 1983 [*confidence gap*].
14. Lipset, Seymour Martin. *Consensus and Conflict: Essays in Political Sociology*. New Brunswick: Transaction Books, 1985 [*other*].
15. Lipset, Seymour Martin. *Continental Divide: The Values and Institutions of the United States and Canada*. New York: Routledge, 1990 [*American exceptionalism*].
16. Lipset, Seymour Martin, and Earl Raab. *Jews and the New American Scene*. Cambridge, Mass: Harvard University Press, 1995 [*jews*].
17. Lipset, Seymour Martin. *American Exceptionalism: A Double-Edged Sword*. New York: W.W. Norton, 1996 [*American exceptionalism*].
18. Lipset, Seymour Martin, and Gary Marks. *It Didn’t Happen Here: Why Socialism Failed in the United States*. New York: W.W. Norton & Co, 2000 [*American exceptionalism/class politics/democracy/extremism*].

*journal articles (in chronological order); 1254,5 weighted pages*

1. Lipset, Seymour Martin. “The Rural Community and Political Leadership in Saskatchewan.” *Canadian Journal of Economics and Political Science* 13, no. 3 (1947): 410–28 [*agrarian socialism/democracy*].
2. Lipset, Seymour Martin. “Political Participation and the Organization of the Cooperative Commonwealth Federation in Saskatchewan.” *Canadian Journal of Economics and Political Science* 14, no. 2 (1948): 191–208 [*agrarian socialism/democracy*].
3. Lipset, Seymour Martin. “Polling and Science.” *The Canadian Journal of Economics and Political Science* 15, no. 2 (1949): 237–40 [*elections*].
4. Lipset, Seymour Martin. “Polling and Science II.” *The Canadian Journal of Economics and Political Science* 16, no. 3 (1950): 426–29 [*elections*].
5. Lipset, Seymour Martin, and Reinhard Bendix. “Social Status and Social Structure: A Re-Examination of Data and Interpretations: I.” *The British Journal of Sociology* 2, no. 2 (1951): 150–68 [*social stratification*].
6. Lipset, Seymour Martin, and Reinhard Bendix. “Social Status and Social Structure: A Re-Examination of Data and Interpretations: II.” *The British Journal of Sociology* 2, no. 3 (1951): 230–54 [*social stratification*].
7. Lipset, Seymour M., and Reinhard Bendix. “Social Mobility and Occupational Career Patterns I. Stability of Jobholding.” *American Journal of Sociology* 57, no. 4 (1952): 366–74 [*social stratification*].
8. Lipset, Seymour M., and Reinhard Bendix. “Social Mobility and Occupational Career Patterns II. Social Mobility.” *American Journal of Sociology* 57, no. 5 (1952): 494–504 [*social stratification*].
9. Lipset, Seymour M. “Democracy in Private Government (A Case Study of the International Typographical Union).” *The British Journal of Sociology* 3, no. 1 (1952): 47–63 [*democracy/trade unions*].
10. Bell, Daniel, and Seymour Martin Lipset. “Introduction.” *Journal of Social Issues* 9, no. 1 (1953): 2–6 [*other*].
11. Lipset, Seymour Martin. “Opinion Formation in a Crisis Situation.” *The Public Opinion Quarterly* 17, no. 1 (1953): 20–46 [*other*].
12. Bendix, Reinhard, Seymour Martin Lipset, and F. Theodore Malm. “Social Origins and Occupational Career Patterns.” *Industrial and Labor Relations Review* 7, no. 2 (1954): 246–61 [*social stratification*].
13. Lipset, Seymour Martin, Reinhard Bendix, and F. Theodore Malm. “Job Plans and Entry into the Labor Market.” *Social Forces* 33, no. 3 (1955): 224–32 [*social stratification*].
14. Lipset, Seymour Martin, and F. Theodore Malm. “First Jobs and Career Patterns.” *American Journal of Economics and Sociology* 14, no. 3 (1955): 247–61 [*social stratification*].
15. Lipset, Seymour Martin. “The Radical Right: A Problem for American Democracy.” *The British Journal of Sociology* 6, no. 2 (1955): 176–209 [*democracy/elections*].
16. Lipset, Seymour Martin. “Jewish Sociologists and Sociologists of the Jews.” *Jewish Social Studies* 17, no. 3 (1955): 177–78 [*jews*].
17. Bendix, Reinhard, and Seymour M. Lipset. “Political Sociology: An Essay with Special Reference to the Development of Research in the United States of America and Western Europe.” *Current Sociology* 6, no. 2 (1957): 79–99 [*other*].
18. Lipset, Seymour Martin. “A Sociologist Looks at History.” *The Pacific Sociological Review* 1, no. 1 (1958): 13–17 [*other*].
19. Lipset, Seymour M., and Hans L. Zetterberg. “A Comparative Study of Social Mobility, Its Causes and Consequences.” *Political Research, Organization and Design* 2, no. 1 (S1958): 7–11 [*social stratification*].
20. Lipset, Seymour Martin. “Some Social Requisites of Democracy: Economic Development and Political Legitimacy.” *American Political Science Review* 53, no. 1 (1959): 69–105 [*American exceptionalism/democracy*].
21. Lipset, Seymour Martin. “American Intellectuals: Their Politics and Status.” *Daedalus* 88, no. 3 (1959): 460–86 [*intellectuals*].
22. Lipset, Seymour Martin. “Democracy and Working-Class Authoritarianism.” *American Sociological Review* 24, no. 4 (1959): 482–501 [*democracy/extremism*].
23. Lipset, Seymour Martin. “Social Stratification and ‘Right-Wing Extremism.’” *The British Journal of Sociology* 10, no. 4 (1959): 346–82 [*democracy/extremism*].
24. Lipset, Seymour Martin. “The Political Animal: Genus Americana.” *Public Opinion Quarterly* 23, no. 4 (1959): 554–62 [*democracy*].
25. Lipset, Seymour Martin. “Party Systems and the Representation of Social Groups.” *European Journal of Sociology* 1, no. 1 (1960): 50–85 [*democracy*].
26. Lipset, Seymour Martin. “‘Working-Class Authoritarianism’: A Reply to Miller and Riessman.” *The British Journal of Sociology* 12, no. 3 (1961): 277–81 [*extremism*].
27. Lipset, Seymour Martin, and Neil Smelser. “Change and Controversy in Recent American Sociology.” *The British Journal of Sociology* 12, no. 1 (1961): 41–51 [*American sociology*].
28. Lipset, Seymour Martin. “Trade Unions and Social Structure: I.” *Industrial Relations* 1, no. 1 (1961): 75–89 [*American exceptionalism/trade unions*]
29. Lipset, Seymour Martin. “Trade Unions and Social Structure: II.” *Industrial Relations* 1, no. 2 (1962): 89–110 [*class politics/trade unions*].
30. Lipset, Seymour Martin. “‘Ideology and Political Bias’: A Reply to Peck.” *The American Catholic Sociological Review* 23, no. 3 (1962): 207–23 [*other*].
31. Lipset, Seymour Martin. “The Value Patterns of Democracy: A Case Study in Comparative Analysis.” *American Sociological Review* 28, no. 4 (1963): 515–31 [*American exceptionalism*]
32. Lipset, Seymour Martin. “Approaches Toward Reducing the Costs of Comparative Survey Research.” *Social Sciences Information* 2, no. 4 (1963): 33–38 [*other*]
33. Lipset, Seymour Martin. “University Students and Politics in Underdeveloped Countries.” *Minerva* 3, no. 1 (1964): 15–56 [*student politics*]
34. Lipset, Seymour Martin. “Canada and the United States-A Comparative View.” *Canadian Review of Sociology* 1, no. 4 (1964): 173–85 [*American exceptionalism/democracy*]
35. Lipset, Seymour Martin. “The Changing Class Structure and Contemporary European Politics.” *Daedalus* 93, no. 1 (1964): 217–303 [*class politics*]
36. Lipset, Seymour Martin. “The Political Behaviour of University Students In Developing Nations.” *Social and Economic Studies* 14, no. 1 (1965): 35–75 [*student politics*]
37. Lipset, Seymour Martin, and Irving Louis Horowitz. “The Birth and Meaning of America: A Discussion of the First New Nation.” *The Sociological Quarterly* 7, no. 1 (1966): 3–20 [*American exceptionalism*]
38. Lipset, Seymour Martin. “Student Opposition in the United States.” *Government and Opposition* 1, no. 3 (1966): 351–74 [*student politics*].
39. Lipset, Seymour Martin. “Students and Politics in Comparative Perspective.” *Daedalus* 97, no. 1 (1968): 1–20 [*politics of academics/student politics*]
40. Ladd, Everett Carll, and Seymour Martin Lipset. “The Politics of American Political Scientists.” *PS: Political Science & Politics* 4, no. 2 (1971): 135–44 [*intellectuals/politics of academics/student politics*].
41. Ladd, Everett Carll, and Seymour Martin Lipset. “American Social Scientists and the Growth of Campus Political Activism in the 1960’s.” *Social Science Information* 10, no. 2 (1971): 105–20 [*politics of academics/student politics*]
42. Lipset, Seymour Martin, and Everett C. Ladd. “The Divided Professoriate.” *Change* 3, no. 3 (1971): 54–60 [*politics of academics*]
43. Lipset, Seymour Martin, and Everett C. Ladd. “The Politics of American Sociologists.” *American Journal of Sociology* 78, no. 1 (1972): 67–104 [*politics of academics*].
44. Lipset, Seymour Martin, and Richard B. Dobson. “The Intellectual as Critic and Rebel: With Special Reference to the United States and the Soviet Union.” *Daedalus* 101, no. 3 (1972): 137–98 [*intellectuals*].
45. Lipset, Seymour Martin. “Ideology and Mythology: Reply to Coleman Romalis (and Other Critics).” *Sociological Inquiry* 42, no. 3–4 (1972): 233–65 [*other*].
46. Ladd, Everett C., and Seymour M. Lipset. “Politics of Academic Natural Scientists and Engineers.” *Science* 176, no. 4039 (1972): 1091–1100 [*politics of academics*].
47. Ladd, Everett C., and Seymour Martin Lipset. “Unionizing the Professoriate.” *Change* 5, no. 6 (1973): 38–44 [*politics of academics*].
48. Lipset, Seymour Martin. “Tom Marshall-Man of Wisdom.” *The British Journal of Sociology* 24, no. 4 (1973): 409–17 [*other*].
49. Lipset, Seymour Martin, and Everett C. Ladd. “Gouldner and ‘The Politics of American Sociologists.’” *American Journal of Sociology* 78, no. 6 (1973): 1485–93 [*other*].
50. Lipset, Seymour Martin. “Education and Equality: Israel and the United States Compared.” *Society* 11, no. 3 (1974): 56–66 [*equality*].
51. Lipset, Seymour Martin, and Everett C. Ladd. “The Myth of the ‘Conservative’ Professor: A Reply to Michael Faia.” *Sociology of Education* 47, no. 2 (1974): 203–13 [*politics of academics*].
52. Lipset, Seymour Martin. “The New Class and the Professoriate.” *Society* 16 (1979): 31–38 [*intellectuals/politics of academics*].
53. Lipset, Seymour Martin. “The Academic Mind at the Top: The Political Behavior and Values of Faculty Elites.” *Public Opinion Quarterly* 46, no. 2 (1982): 143–68 [*politics of academics*].
54. Lipset, Seymour Martin. “Democracy at the Polls: An Expository Review.” *Electoral Studies* 1, no. 1 (1982): 107–15 [*democracy/elections*].
55. Lipset, Seymour Martin. “Radicalism or Reformism: The Sources of Working-Class Politics.” *American Political Science Review* 77, no. 1 (1983): 1–18 [*class politics/democracy/extremism*].
56. Lipset, Seymour Martin, and William Schneider. “The Decline of Confidence in American Institutions.” *Political Science Quarterly* 98, no. 3 (1983): 379-402 [*confidence gap*].
57. Lipset, Seymour Martin. “China in Transition: A Travel Memoir, May-June, 1984.” *PS: Political Science & Politics* 17, no. 4 (1984): 765–77 [*other*].
58. Lipset, Seymour Martin. “Historical Traditions and National Characteristics: A Comparative Analysis of Canada and the United States.” *Canadian Journal of Sociology* 11, no. 2 (1986): 113–55 [*American exceptionalism*].
59. Lipset, Seymour Martin. “The Elections, the Economy and Public Opinion: 1984.” *PS: Political Science & Politics* 18, no. 1 (1985): 28–38 [*elections*].
60. Lipset, Seymour Martin. “Beyond 1984: The Anomalies of American Politics.” *PS: Political Science & Politics* 19, no. 2 (1986): 222–36 [*elections*].
61. Lipset, Seymour Martin, and William Schneider. “The Confidence Gap during the Reagan Years, 1981-1987.” *Political Science Quarterly* 102, no. 1 (1987): 1–23 [*confidence gap*].
62. Lipset, Seymour Martin. “Comparing Canadian and American Unions.” *Society* 24, no. 2 (1987): 60–70 [*American exceptionalism/trade unions*].
63. Diamond, Larry, Seymour Martin Lipset, and Juan Linz. “Building and Sustaining Democratic Government in Developing Countries: Some Tentative Findings.” *World Affairs* 150, no. 1 (1987): 5–19 [*democracy*].
64. Lipset, Seymour Martin. “Neoconservatism: Myth and Reality.” *Society* 25, no. 5 (1988): 29–37 [i*ntellectuals*].
65. Lipset, Seymour Martin. “The US Elections: The Status Quo Re-Affirmed.” *International Journal of Public Opinion Research* 1, no. 1 (1989): 25–44 [*democracy/elections*].
66. Lipset, Seymour Martin. “Voluntary Activities: More Canadian-American Comparisons: A Reply.” *Canadian Journal of Sociology* 14, no. 3 (1989): 377–82 [*American exceptionalism*].
67. Lipset, Seymour Martin. “Politics and Society in the USSR: A Traveller’s Report.” *PS: Political Science and Politics* 23, no. 1 (1990): 20–28 [*other*].
68. Lipset, Seymour Martin. “A Unique People in an Exceptional Country.” *Society* 28, no. 2 (1990): 3–13 [*American exceptionalism/jews*].
69. Lipset, Seymour Martin. “The Values of Canadians and Americans: A Reply.” *Social Forces* 69, no. 1 (1990): 267–72 [*American exceptionalism*].
70. Lipset, Seymour Martin. “The Centrality of Political Culture.” *Journal of Democracy* 1, no. 4 (1990): 80–83 [*American exceptionalism/democracy*].
71. Lipset, Seymour Martin. “Trade Unionism in Canada and the United States: A Reply to Bowden.” *Canadian Review of Sociology* 27, no. 4 (1990): 531–35 [*American exceptionalism/democracy/trade unions*].
72. Clark, Terry Nichols, and Seymour Martin Lipset. “Are Social Classes Dying?” *International Sociology* 6, no. 4 (1991): 397–410 [*social stratification*].
73. Lipset, Seymour Martin. “Equal Chances versus Equal Rights.” *The Annals of the American Academy of Political and Social Science* 523 (1992): 63–74 [*equality*].
74. Jalali, Rita, and Seymour Martin Lipset. “Racial and Ethnic Conflicts: A Global Perspective.” *Political Science Quarterly* 107, no. 4 (1992): 585-606 [*other*].
75. Lipset, Seymour Martin. “The Work Ethic, Then and Now.” *Journal of Labor Research* 13, no. 1 (1992): 45–54 [*other*].
76. Lipset, Seymour Martin. “The Significance of the 1992 Election.” *PS: Political Science and Politics* 26, no. 1 (1993): 7-16 [*elections*].
77. Lipset, Seymour Martin. “Reflections on Capitalism, Socialism & Democracy.” *Journal of Democracy* 4, no. 2 (1993): 43–55 [*American exceptionalism/democracy*].
78. Lipset, Seymour Martin. “Roosevelt and Clinton.” *Society* 30, no. 3 (1993): 73–76 [*elections*].
79. Lipset, Seymour Martin. “Culture and Economic Behavior: A Commentary.” *Journal of Labor Economics* 11, no. 1 (1993): 330–47 [*American exceptionalism*].
80. Clark, Terry Nichols, Seymour Martin Lipset, and Michael Rempel. “The Declining Political Significance of Class.” *International Sociology* 8, no. 3 (1993): 293–316 [*class politics/extremism*].
81. Lipset, Seymour Martin. “Pacific Divide: American Exceptionalism – Japanese Uniqueness.” *International Journal of Public Opinion Research* 5, no. 2 (1993): 121–66 [*American exceptionalism*].
82. Lipset, Seymour Martin, and Gyorgy Bence. “Anticipations of the Failure of Communism.” *Theory and Society* 23, no. 2 (1994): 169–210 [other].
83. Lipset, Seymour Martin. “The State of American Sociology.” *Sociological Forum* 9, no. 2 (1994): 199–220 [American sociology/politics of academics].
84. Lipset, Seymour Martin. “The Social Requisites of Democracy Revisited: 1993 Presidential Address.” *American Sociological Review* 59, no. 1 (1994): 1–22 [democracy].
85. Lipset, Seymour Martin. “Trade Union Exceptionalism: The United States and Canada.” *The ANNALS of the American Academy of Political and Social Science* 538 (1995): 115–30 [*American exceptionalism/trade unions*].
86. Lipset, Seymour Martin. “Malaise and Resiliency in America.” *Journal of Democracy* 6, no. 3 (1995): 4–18 [*American exceptionalism/democracy*].
87. Lipset, Seymour Martin. “Steady Work: An Academic Memoir.” *Annual Review of Sociology* 22, no. 1 (1996): 1–27 [*American exceptionalism/democracy*].
88. Lipset, Seymour Martin, and Marcella R. Ray. “Technology, Work, and Social Change.” *Journal of Labor Research* 17, no. 4 (1996): 613–26 [*other*].
89. Lipset, Seymour Martin. “American Union Density in Comparative Perspective.” *Contemporary Sociology* 27, no. 2 (1998): 123–25 [*American exceptionalism/trade unions*].
90. Lipset, Seymour Martin. “Failures of Extremism.” *Society* 35, no. 2 (1998): 245–57 [*other*].
91. Lipset, Seymour Martin, and Ivan Katchanovski. “The Future of Private Sector Unions in the U.S.” *Journal of Labor Research* 22, no. 2 (2001): 229–44 [*trade unions*].
92. Lipset, Seymour Martin. “The Americanization of the European Left.” *Journal of Democracy* 12, no. 2 (2001): 74–87 [*American exceptionalism/democracy*].
93. Rothman, Stanley, Seymour Martin Lipset, and Neil Nevitte. “Does Enrollment Diversity Improve University Education?” *International Journal of Public Opinion Research* 15, no. 1 (2003): 8–26 [*other*].

**A2. Edited books considered in the citation analyses**

1. Lipset, Seymour Martin, and Walter Galenson, eds. *Labor and Trade Unionism*. New York: John Wiley, 1960.
2. Lipset, Seymour Martin, and Neil J. Smelser, eds. *Sociology: The Progress of a Decade*. Englewood Cliffs, N.J.: Prentice-Hall, 1961.
3. Lipset, Seymour Martin, and Leo Lowenthal, eds. *Culture and Social Character. The Work of David Riesman Reviewed*. New York: Free Press, 1961.
4. Lipset, Seymour Martin, ed. *Democracy and the Organization of Political Parties (Abridged Modern Ed. Of M. Ostrogorski)*. Garden City, N.Y.: Doubleday, 1964.
5. Lipset, Seymour Martin, and Sheldon S. Wolin, eds. *The Berkeley Student Revolt: Facts and Interpretations*. Garden City, N.Y.: Anchor Books, 1965.
6. Bendix, Reinhard, and Seymour Martin Lipset, eds. *Class, Status and Power. Social Stratification in Comparative Perspective*. New York: The Free Press, 1966.
7. Smelser, Neil J., and Seymour Martin Lipset, eds. *Social Structure and Mobility in Economic Development*. Chicago: Aldine Publishing Co., 1966.
8. Lipset, Seymour Martin, and Aldo Solari, eds. *Elites in Latin America*. New York: Oxford University Press, 1967.
9. Lipset, Seymour Martin, and Stein Rokkan, eds. *Party Systems and Voter Alignments: Cross-National Perspectives*. New York: The Free Press, 1967.
10. Lipset, Seymour Martin, ed. *Student Politics*. New York: Basic Books, 1967.
11. Lipset, Seymour Martin, and Richard Hofstadter, eds. *Sociology and History: Methods*. New York and London: Basic Books, 1968.
12. Lipset, Seymour Martin, ed. *Politics and the Social Sciences*. New York: Oxford University Press, 1969.
13. Lipset, Seymour Martin, and Philip G. Altbach, eds. *Students in Revolt*. Boston: Houghton Mifflin Company, 1969.
14. Laslett, John H. M., and Seymour Martin Lipset, eds. *Failure of a Dream? Essays in the History of American Socialism*. Garden City, N.Y.: Doubleday, 1974.
15. Lipset, Seymour Martin, ed. *Emerging Coalitions in American Politics*. San Francisco: Institute for Contemporary Studies, 1978.
16. Lipset, Seymour Martin, ed. *The Third Century: America as a Post-Industrial Society*. A Phoenix Book. Chicago: The University of Chicago Press, 1980.
17. Lipset, Seymour Martin, ed. *Unions in Transition: Entering the Second Century*. San Francisco: ICS, 1986.
18. Larry Jay Diamond, Juan J. Linz, and Seymour Martin Lipset, eds. *Democracy in Developing Countries (3 Vols)*. Boulder: Lynne Rienner Publishers, 1988.
19. Lipset, Seymour Martin, ed. *American Pluralism and the Jewish Community*. New Brunswick, N.J: Transaction Publishers, 1990.
20. Diamond, Larry, Juan J. Linz, and Seymour Martin Lipset, eds. *Politics in Developing Countries: Comparing Experiences with Democracy*. Boulder: Lynne Rienner Publishers, 1995.
21. Lipset, Seymour Martin, ed. *The Encyclopedia of Democracy*. London: Routledge, 1995.
22. Lipset, Seymour Martin, ed. *Democracy in Europe and the Americas*. Washington, DC: Congressional Quarterly, 1998.
23. Clark, Terry Nichols, and Seymour Martin Lipset, eds. *The Breakdown of Class Politics: A Debate on Post-Industrial Stratification*. Baltimore: Johns Hopkins University Press, 2001.

**A3. Dictionaries**

| **agrarian**  **socialism** | **American**  **exceptionalism** | **American**  **sociology** | **class**  **politics** | **confidence**  **gap** |
| --- | --- | --- | --- | --- |
| canada | america | American | behavior | attitude |
| co-operativ | canada | ASA | class | business |
| communit | canadian | Association | communist | companies |
| constituenc | catholic | Berkeley | countries | confidence |
| delegate | church | Chicago | economic | decline |
| economic | comparativ | Columbia | france | gallup |
| farmer | counterrevolution | department | germany | gap |
| government | countr | disciplin | ideology | government |
| grain | culture | Harvard | italy | industry |
| leader | democracy | Lazarsfeld | labor | institution |
| member | democratic | Lynd | modernization | labor |
| movement | difference | Merton | movement | leader |
| organization | equality | Mills | parties | national |
| party | exceptional | Parsons | party | opinion |
| people | french | scholar | politic | political |
| provinc | government | scienc | socialist | poll |
| province | group | social | strata | public |
| rural | history | sociolog | support | question |
| saskatchewan | immigrant | Sorokin | trade | regulation |
| work | individual | theory | traditional | respondent |
|  | institution |  | union | society |
|  | nation |  | vot | survey |
|  | New |  | work | trust |
|  | north |  |  | year |
|  | people |  |  |  |
|  | Quebec |  |  |  |
|  | religio |  |  |  |
|  | religious |  |  |  |
|  | respect |  |  |  |
|  | revolution |  |  |  |
|  | rights |  |  |  |
|  | socialism |  |  |  |
|  | socialist |  |  |  |
|  | society |  |  |  |
|  | states |  |  |  |
|  | system |  |  |  |
|  | Toronto |  |  |  |
|  | united |  |  |  |
|  | value |  |  |  |
|  | variation |  |  |  |
|  | War |  |  |  |
|  | welfare |  |  |  |
|  | York |  |  |  |

*Notes***.** All words are not case-sensitive and can match either completely *or* partly. Thus the string of character ‘scienc’ codes the following words: science, scientific, scientist, scientists, etc.

| **democracy** | **elections** | **equality** | **extremism** | **intellectuals** |
| --- | --- | --- | --- | --- |
| american | Bush | action | anti-semit | academic |
| behavior | campaign | affirmative | authoritar | america |
| class | candidate | america | catholic | critic |
| condition | Carter | arab | class | cultural |
| conflict | Clinton | background | communist | french |
| constitution | congress | black | conservat | group |
| control | democrat | education | extremis | ideological |
| democrac | election | equal | fascism | intellectual |
| democrat | electorate | famil | group | intelligentsia |
| development | government | group | Hitler | left |
| dictatorship | majority | high | ideolog | liberal |
| economic | opinion | income | lower | mass |
| election | parties | israel | McCarthy | occupation |
| electora | Party | opportunity | middle | opinion |
| government | people | political | minorit | party |
| group | policies | rights | movement | political |
| institution | political | school | national | public |
| leader | Poll | society | nazi | radical |
| legitimac | pollster | white | parties | rebel |
| member | president |  | party | scholar |
| middle | public |  | political | society |
| movement | Reagan |  | psychological | soviet |
| opposition | republican |  | radical | states |
| organization | Roosevelt |  | religio | united |
| participation | support |  | revolution | value |
| parties | Vote |  | scapegoat | writer |
| party |  |  | social | york |
| power |  |  | support |  |
| representation |  |  | traditional |  |
| requisites |  |  | vote |  |
| revolution |  |  | workers |  |
| rights |  |  |  |  |
| society |  |  |  |  |
| stable |  |  |  |  |
| states |  |  |  |  |
| strata |  |  |  |  |
| support |  |  |  |  |
| system |  |  |  |  |
| united |  |  |  |  |
| vot |  |  |  |  |
| worker |  |  |  |  |
|  |  |  |  |  |
|  |  |  |  |  |
|  |  |  |  |  |
|  |  |  |  |  |
|  |  |  |  |  |
|  |  |  |  |  |

| **jews** | **politics of**  **academics** | **social**  **stratification** | **student**  **politics** | **trade**  **unions** |
| --- | --- | --- | --- | --- |
| america | academ | business | activism | american |
| antisemi | campus | career | activit | bargaining |
| communit | carnegie | children | attitud | behavior |
| group | colleag | class | behavior | business |
| jew | colleg | data | Berkeley | Canada |
| nazi | committee | division | college | culture |
| religio | conservativ | economic | communist | democratic |
| society | controvers | education | generation | density |
| sociolog | Data | famil | graduat | government |
| states | department | father | left | industrial |
| tribal | discipline | hierarch | movement | international |
| united | educat | incomes | opposition | labor |
|  | Elite | job | parties | law |
|  | engineer | labor | party | leader |
|  | faculty | manual | politic | member |
|  | Field | Marx | protest | movement |
|  | harvard | mobility | radical | organization |
|  | intellectual | nonmanual | revolution | power |
|  | Issue | occupat | student | private |
|  | Left | position | survey | public |
|  | liberal | power | underdeveloped | skilled |
|  | member | respondent | university | trade |
|  | opinion | status | vietnam | typo |
|  | orientation | stratificat | view | union |
|  | politic | structur | young | values |
|  | president | unskill | youth | wage |
|  | profession | Warner |  | worker |
|  | professor | white-col |  |  |
|  | scholar | work |  |  |
|  | science |  |  |  |
|  | scient |  |  |  |
|  | subject |  |  |  |
|  | survey |  |  |  |
|  | Teach |  |  |  |
|  | universit |  |  |  |
|  | Value |  |  |  |
|  | View |  |  |  |
|  |  |  |  |  |
|  |  |  |  |  |
|  |  |  |  |  |
|  |  |  |  |  |
|  |  |  |  |  |
|  |  |  |  |  |
|  |  |  |  |  |
|  |  |  |  |  |
|  |  |  |  |  |
|  |  |  |  |  |

**A4. Five most cited contributions in the SSCI by publication type and WoS category**

*Political Science: authored books*

| book title (abbreviation) | no. of citing journal articles | % of all citing journal articles |
| --- | --- | --- |
| Political Man | 1,501 | 60,0 |
| The First New Nation | 176 | 7,1 |
| The Confidence Gap | 133 | 5,4 |
| American Exceptionalism | 114 | 4,6 |
| Union Democracy | 101 | 4,1 |
| all authored books | 2,465 | 100 |

*Political Science: edited books*

| book title (abbreviation) | no. of citing journal articles | % of all citing journal articles |
| --- | --- | --- |
| Party Systems and Voter Alignment | 1,210 | 70,1 |
| Democracy in Developing Countries | 221 | 12,8 |
| Class Status Power | 52 | 3,0 |
| Politics in Developing Countries | 44 | 2,5 |
| Elites in Latin America | 38 | 2,2 |
| all edited books | 1,726 | 100 |

*Political Science: journal articles*

| journal article title (abbreviation) | no. of citing journal articles | % of all citing journal articles |
| --- | --- | --- |
| Some Social Requisites of Democracy | 675 | 54.0 |
| The Social Requisites of Democracy Revisited | 131 | 10,5 |
| Democracy and Working-Class Authoritarianism | 46 | 3,7 |
| Comparative Analysis of the Social Requisites of Democracy | 43 | 3,4 |
| Are Social Classes Dying? | 36 | 2,9 |
| all journal articles | 1,249 | 100 |

*Sociology: authored books*

| book title (abbreviation) | no. of citing journal articles | % of all citing journal articles |
| --- | --- | --- |
| Political Man | 886 | 33,5 |
| Social Mobility in Industrial Society | 556 | 21,1 |
| Union Democracy | 250 | 9,5 |
| The First New Nation | 211 | 8,0 |
| Politics of Unreason | 118 | 4,5 |
| all authored books | 2,644 | 100 |

*Sociology: edited books*

| book title (abbreviation) | no. of citing journal articles | % of all citing journal articles |
| --- | --- | --- |
| Party Systems and Voter Alignment | 243 | 33,9 |
| Class, Status and Power | 181 | 25,3 |
| Social Structure, Mobility and Development | 46 | 6,4 |
| Breakdown of Class Politics | 29 | 4,0 |
| Berkeley Student Revolt | 28 | 4,0 |
| all edited books | 716 | 100 |

*Sociology: journal articles*

| journal article title (abbreviation) | no. of citing journal articles | % of all citing journal articles |
| --- | --- | --- |
| Some Social Requisites of Democracy | 127 | 15,6 |
| Are Social Classes Dying? | 95 | 11,7 |
| The Social Requisites of Democracy Revisited | 52 | 6,4 |
| Democracy and Working-Class Authoritarianism | 49 | 6,0 |
| The Value Patterns of Democracy | 44 | 5,4 |
| all journal articles | 815 | 100 |

1. Assigned topics are indicated in brackets. [↑](#footnote-ref-1)
